# Extracorporeal membrane oxygenation for COVID-19 and influenza H1N1 associated acute respiratory distress syndrome: a multicenter retrospective cohort study

**DOI:** 10.1186/s13054-022-03906-4

**Published:** 2022-02-05

**Authors:** Vito Fanelli, Marco Giani, Giacomo Grasselli, Francesco Mojoli, Gennaro Martucci, Lorenzo Grazioli, Francesco Alessandri, Silvia Mongodi, Gabriele Sales, Giorgia Montrucchio, Costanza Pizzi, Lorenzo Richiardi, Luca Lorini, Antonio Arcadipane, Antonio Pesenti, Giuseppe Foti, Nicolò Patroniti, Luca Brazzi, VMarco Ranieri

**Affiliations:** 1 Department of Surgical Sciences, University of Turin, Turin, Italy; 2 Department of Anaesthesia, Critical Care and Emergency - Città Della Salute E Della Scienza Hospital, University of Turin, Corso Dogliotti 14, 10126 Turin, Italy; 3 Department of Emergency and Intensive Care, School of Medicine and Surgery, ASST Monza, University of Milano-Bicocca, Monza, Italy; 4 Department of Anesthesia, Critical Care and Emergency, Fondazione IRCCS Ca’ Granda Ospedale Maggiore Policlinico, Milan, Italy; 5 Anesthesia and Intensive Care, Fondazione IRCCS Policlinico San Matteo, Università Degli Studi Di Pavia, Pavia, Italy; 6 Department of Anesthesia and Intensive Care, IRCCS-ISMETT (Istituto Mediterraneo Per I Trapianti E Terapie Ad Alta Specializzazione), Palermo, Italy; 7 Department Emergency and Critical Area, ASST Papa Giovanni XXIII, Bergamo, Italy; 8 Department of Anesthesia and Intensive Care Medicine, Sapienza” University of Rome, Policlinico Umberto I, Rome, Italy; 9 Department of Medical Sciences, University of Turin, Turin, Italy; 10 Cancer Epidemiology Unit, Città Della Salute E Della Scienza Di Torino University Hospital and CPO-Piemonte, Turin, Italy; 11 Anesthesia and Intensive Care,, San Martino Policlinico Hospital - IRCCS for Oncology and Neurosciences, Genoa, Italy; 12 Department of Surgical Sciences and Integrated Diagnostics [DISC], University of Genoa, Genoa, Italy; 13 Alma Mater Studiorum, Dipartimento Di Scienze Mediche E Chirurgiche (DIMEC), Anesthesia and Intensive Care Medicine, Università Di Bologna, IRCCS Policlinico Di Sant’Orsola, Bologna, Italy

**Keywords:** COVID-19, H1N1, Influenza, ARDS, ECMO

## Abstract

**Background:**

Extracorporeal membrane oxygenation (ECMO) has become an established rescue therapy for severe acute respiratory distress syndrome (ARDS) in several etiologies including influenza A H1N1 pneumonia. The benefit of receiving ECMO in coronavirus disease 2019 (COVID-19) is still uncertain. The aim of this analysis was to compare the outcome of patients who received veno-venous ECMO for COVID-19 and Influenza A H1N1 associated ARDS.

**Methods:**

This was a multicenter retrospective cohort study including adults with ARDS, receiving ECMO for COVID-19 and influenza A H1N1 pneumonia between 2009 and 2021 in seven Italian ICU. The primary outcome was any-cause mortality at 60 days after ECMO initiation. We used a multivariable Cox model to estimate the difference in mortality accounting for patients’ characteristics and treatment factors before ECMO was started. Secondary outcomes were mortality at 90 days, ICU and hospital length of stay and ECMO associated complications.

**Results:**

Data from 308 patients with COVID-19 (*N* = 146) and H1N1 (*N* = 162) associated ARDS who had received ECMO support were included. The estimated cumulative mortality at 60 days after initiating ECMO was higher in COVID-19 (46%) than H1N1 (27%) patients (hazard ratio 1.76, 95% CI 1.17–2.46). When adjusting for confounders, specifically age and hospital length of stay before ECMO support, the hazard ratio decreased to 1.39, 95% CI 0.78–2.47. ICU and hospital length of stay, duration of ECMO and invasive mechanical ventilation and ECMO-associated hemorrhagic complications were higher in COVID-19 than H1N1 patients.

**Conclusion:**

In patients with ARDS who received ECMO, the observed unadjusted 60-day mortality was higher in cases of COVID-19 than H1N1 pneumonia. This difference in mortality was not significant after multivariable adjustment; older age and longer hospital length of stay before ECMO emerged as important covariates that could explain the observed difference.

*Trial registration number*: NCT05080933, retrospectively registered.

**Supplementary Information:**

The online version contains supplementary material available at 10.1186/s13054-022-03906-4.

## Introduction

Since the early 2000s, the critical care community experienced several waves of Acute Respiratory Distress Syndrome (ARDS)-associated pneumonia caused by virus infection that challenged the capacity of health care systems to provide high quality care for these critically ill patients [1–4]. Veno-venous extracorporeal membrane oxygenation (VV ECMO) has been used as a rescue supportive treatment in ARDS-associated viral pneumonia during influenza A H1N1 pandemia with a hospital mortality ranging between 29 and 32% [2, 3, 5]. Recent reports suggest that SARS-CoV-2 infection is more severe than influenza [6]. Hospital mortality, likelihood of receiving invasive mechanical ventilation and ICU length of stay have been shown to be three times higher in COVID-19 patients compared to seasonal influenza [6]. Initial reports on using ECMO for COVID-19 patients were disappointing, with a mortality rate ranging between 65 and 94% [7, 8]. However, in two multicenter studies that enrolled highly selected patients who received the best standard of care at the early stage of the disease, a probability of 60- and 90-day mortality was significantly lower (31 and 38%, respectively) [9, 10], and hospital mortality of 37% was similar to that from robust metanalysis of observational studies and randomized clinical trials examining ECMO in adults with COVID-19 ARDS [11]. Considering these dissimilar results, the present study assessed whether the outcome of patients who received VV-ECMO for COVID-19 and Influenza A H1N1 associated ARDS depends on different viral etiologies.

## Methods

This multi-center retrospective cohort study was conducted at ECMO referral centers of seven Italian teaching hospitals (Additional file 1: Table 1e) from August 22th 2009 to February 28th 2021. The institutional review board of each participating hospital approved the study using data collected for routine clinical practice and waived the requirement for informed consent. We collected data on all consecutive adult patients who were supported with VV ECMO for severe ARDS due to confirmed (real-time RT-PCR on nasopharyngeal swabs, or lower respiratory tract aspirates) COVID-19 and influenza A (H1N1). Centers involved in the study are part of a national ECMO network where specialists perform remote assessment, deliver advice, and consider patients against eligibility criteria for retrieval on mobile ECMO, since the 2009 influenza A H1N1 pandemic [2]. In all centers, patients were considered eligible for VV ECMO according to shared criteria that are shown in the electronic supplementary material. All participating centers contributed to both H1N1 and COVID-19 patient cohorts.

**Table 1:** Baseline characteristics of COVID-19 and H1N1 patients with ARDS

Variables	COVID-19 *N* = 146	H1N1 *N* = 162	*P* value
Age, yrs	53 (48–59)	47 (37–58)	0.0003
Gender—male, *n *(%)	124 (85)	100 (62)	0.0001
BMI	29 (26–34)	29 (25–33)	0.1938
Underlying comorbidities, *n *(%)			
Obesity, *n *(%)	61 (42)	68 (43)	0.825
Arterial hypertension, *n *(%)	73 (50)	44 (27)	0.0001
Smoking, *n *(%)	26 (18)	29 (18)	0.983
Diabetes, *n *(%)	37 (25)	23 (14)	0.014
Asthma, *n *(%)	8 (5)	5 (3)	0.297
COPD*, n (*%*)*	2 (1)	8 (5)	0.078
Pregnancy*, n (%)*	2 (1)	6 (4)	0.199
Chronic heart failure*, n (*%*)*	3 (2)	7 (4)	0.263
Chronic liver disease, *n (*%*)*	4 (3)	2 (1)	0.340
Chronic renal failure, *n (*%*)*	4 (3)	1 (1)	0.141
Malignancy, *n (*%*)*	2 (1)	7 (4)	0.125
Chronic immunosuppression, *n* (*%*)	8 (5)	3 (2)	0.087
SOFA	7 (5–9)	8 (6–11)	0.0017
SAPS II	35 (27–49)	35 (27–47)	0.5591
X-ray quadrants involved, *n*	4 (4–4)	4 (3–4)	0.0004
Hospital days before ECMO	11 (6–17)	4 (2–8)	0.0001
ICU days before ECMO	7 (3–12)	0 (0–3)	0.0001
Days of IMV before ECMO	5(2–9)	2(1–6)	0.0001
Rescue therapies pre-ECMO, *n (%)*			
Lung recruitment maneuvers	104 (65)	88 (73)	0.168
Prone Position	111 (78)	57 (35)	0.0001
Inhaled nitric oxide	51 (36)	24 (15)	0.0001
Ventilation setting and ABG pre-ECMO			
PaO_2_/FiO_2_, mmHg	66 (56–80)	64 (54–82)	0.3373
PaCO_2_, mmHg	59 (51–74)	56 (48–68)	0.0492
pH	7.34 (7.25–7.39)	7.33 (7.28–7.39)	0.9772
FiO_2_	1 (0.95–1)	1 (1–1)	0.0104
PEEP, cmH_2_O	12 (10–14)	15 (14–18)	0.0001
VT/PBW, ml	6 (5–7)	6 (6–7)	0.0237
RR, bpm	25 (22–30)	26 (20–33)	0.0821
Pplat, cmH_2_O	29 (26–31)	31 (29–33)	0.0001
Driving Pressure, cmH_2_O	16 (13–19)	15 (13–18)	0.0515
Compliance respiratory system, ml/cmH_2_O	26 (21–34)	29 (21–36)	0.126

BMI: body mass index. COPD: Chronic obstructive pulmonary disease. SOFA: Sequential Organ Failure Assessment. SAPS II: Simplified Acute Physiology Score II. LRM: Lung Recruitment Maneuvers. NO: nitric oxide. LOS: length of stay. IMV: invasive mechanical ventilation. PaO_2_/FiO_2_: ratio between arterial pressure and inspired fraction of oxygen. PaCO_2_: arterial pressure of carbon dioxide. PEEP: positive end expiratory pressure. VT/PBW: tidal volume divided by predicted body weight. RR: respiratory rate. Pplat: plateau pressure

### Data collection

Baseline characteristics included dates of admission to the hospital and to the referral ICU, age, sex, pregnancy status, medical history, presence of risk factors for complicated influenza, presence of coexisting chronic disease, weight and height used to calculate the body mass index (BMI) and the predicted body weight (PBW), initial severity assessed by the SAPS II score and organ failure assessed by the SOFA score. Before starting ECMO, we recorded the time and duration of hospitalization, of endotracheal intubation, the use of rescue therapies such as prone positioning, inhaled nitric oxide (iNO), lung recruitment maneuvers (LRM), respiratory parameters (i.e. tidal volume, positive end expiratory pressure, plateau pressure, driving pressure, PaO_2_/FiO_2_ ratio), arterial blood gas, and number of lung segments affected on the chest X‐ray. After ECMO cannulation, we recorded respiratory parameters, heparin dose, extracorporeal support operational characteristics (blood flow, sweep gas rate) at day 1, 7, 14, and 21 under ECMO support. Moreover, time and duration of ECMO treatment and hemorrhagic and mechanical complications were recorded. The number of missing data for each variable recorded is shown Additional file 1: Table E1. Patients were followed-up till hospital discharge or death.

### Study outcomes

The primary outcome was in-hospital death in a time-to-event analysis assessed at 60 days after ECMO initiation. Secondary outcomes were in-hospital death in a time-to-event analysis assessed at 90 days after ECMO initiation, duration of ECMO and invasive mechanical ventilation, ICU and length of hospital stay and the occurrence of hemorrhagic complications (cannula insertion site, airways, gastro-intestinal and central nervous system) and mechanical complications (cannula thrombosis, membrane clotting and pump malfunction) while receiving ECMO.

### Statistical analysis

Continuous variables are presented as medians and interquartile ranges (IQR). Categorical variables are presented as counts and percentages. We compared medians and percentages between COVID-19 and H1N1 groups with rank sum and chi square tests, respectively. We estimated the distribution of mortality (for any cause) over 60 days by Kaplan–Meier curves, and we checked if hospital discharge was a competing event by calculating the cumulative incidence function using the Fine and Grey model. We fit a Cox proportional hazards model for the outcomes of 60- and 90-day mortality accounting for patient characteristics and treatment factors before starting ECMO. We censored patients who were still hospitalized at the time of the last database update. The Cox model estimated the hazard ratio of death accounting for the following potential confounders categorized as shown in Table 1: age, sex, body mass index, underlying comorbidities, patient severity (SOFA), pre-ECMO hospital length of stay, rescue therapies before ECMO (lung recruiting maneuvers, prone position and inhaled nitric oxide), ventilation settings and gas exchange before ECMO. The hazard ratio of death at 60 days was also estimated, accounting for potential confounders such as ventilation settings (tidal volume, PEEP, respiratory rate, plateau pressure and driving pressure) and operational characteristics of ECMO (blood flow and sweep gas) at day 1 of extracorporeal support. In addition, the Respiratory ECMO Survival Prediction (RESP)-Score, which is a validated tool to predict survival for patients that receive ECMO for respiratory failure [12], was incorporated into the Cox model either by adding it to the above selected variables or by itself. The hazard ratio of death at 60 and 90 days was estimated, stratifying by center-level. Multiple imputation (50 imputed datasets) was used to account for missing values, using chained equations that fill in missing values in multiple variables iteratively. To further account for potential confounding, the propensity score of being affected by COVID-19 vs H1N1 based on all the covariates described above was calculated using a probit model, and the derived score was treated as covariate in the Cox model as a sensitivity analysis. Only subjects belonging to the common support were included both in the main multivariable Cox model adjusted for all the potential confounders and in the model adjusted for the propensity score. The common support is the area where the estimated propensity scores for COVID-19 and H1N1 patients overlap. A convenient sample size for the study was planned in order to include patients admitted to ICUs of participating centers in the study period (from August 22^th^ 2009 to February 28^th^ 2021). Statistical analyses were performed using Stata 16.1/SE (Stata Corporation, Texas, USA).

## Results

### Baseline characteristics prior to ECMO

Patients’ characteristics at baseline and the number of missing data before VV ECMO initiation in the two groups are reported in Tables 1 and Additional file 1: Table 2e, respectively. COVID-19 patients were older compared to H1N1 patients (median years 53 vs 47; P = 0.0003). Moreover, arterial hypertension (50 vs 27%; P = 0.000) and diabetes (25 vs 14%; P = 0.014) were more frequent in COVID-19 than H1N1 patients. Duration of hospital and ICU length of stay and of invasive mechanical ventilation before ECMO initiation was longer in COVID-19 patients. Rescue therapies, such as prone position (78 vs 35%) and inhaled nitric oxide (36 vs 15%), were applied prior to VV ECMO more frequently in COVID-19 than H1N1 patients. Median PaCO_2_ and driving pressure were higher in COVID-19 than H1N1 patients, while median PEEP was higher in H1N1 than COVID-19 patients.

### Study outcomes

**Fig. 1:**
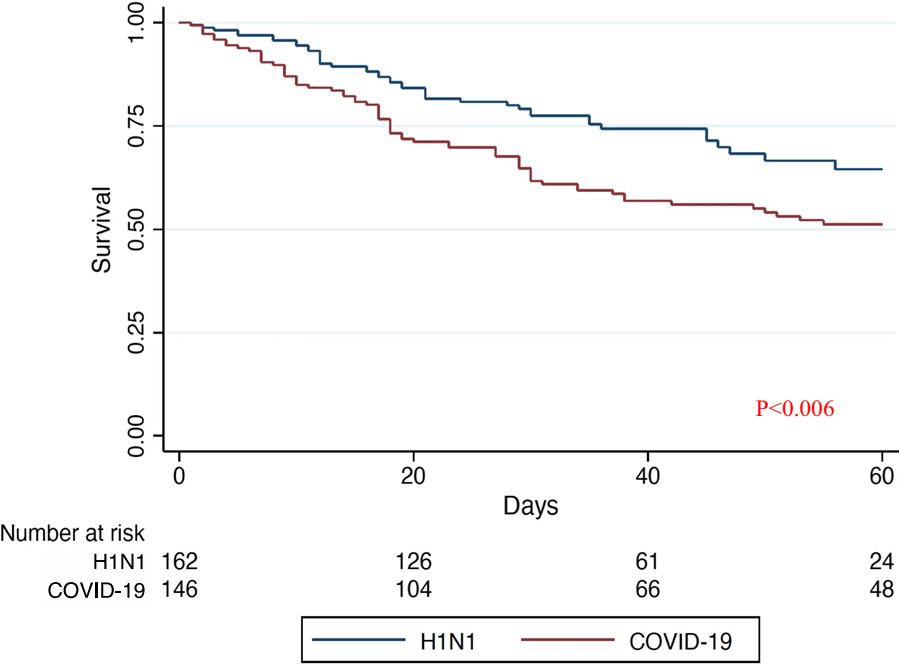
Kaplan-Mayer survival curves for COVID-19 and influenza A H1N1 patients

**Fig. 2:**
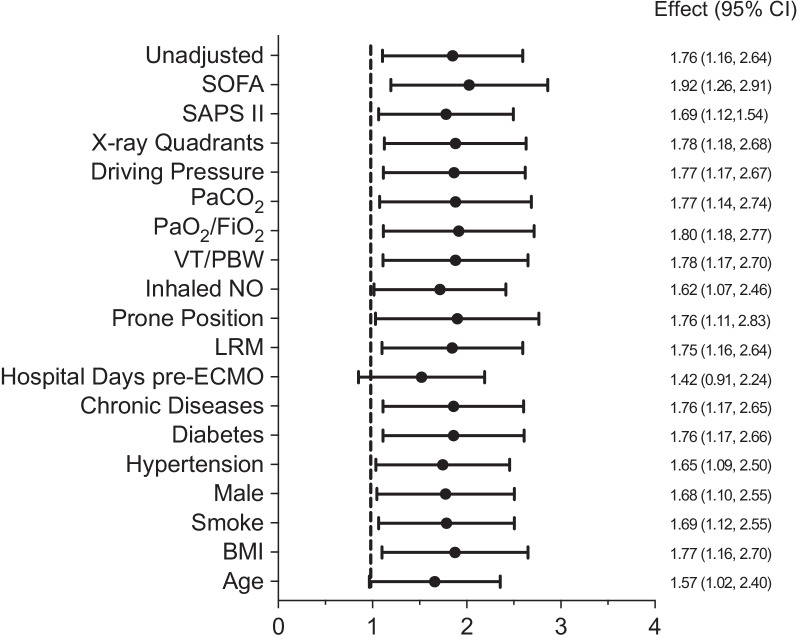
Cox model of factors accounting for differences in 60-day mortality after ECMO in patients with COVID-19 versus influenza A H1N1

At 60 days, 67 patients (46%) in the COVID-19 group and 43 (27%) patients in the H1N1 group had died (Figs. 1 and 1E). Similar results were obtained when hospital discharge was considered as a competing event using the Fine and Gray model. The hazard ratio of death at 60 days stratified per center and estimated using the Cox model was 1.76, 95% CI 1.17–2.64. When adjusting for confounders, mainly age and hospital length of stay before ECMO support (Fig. 2), the hazard ratio decreased to 1.39, 95% CI 0.78–2.47 (Table 2). Similar results were observed when propensity score analysis was used to account for confounders, hazard ratio for mortality of 1.21, 95% CI 0.7–2.09. The adjusted hazard ratios of death at 60 days adding RESP score to previously selected variables or RESP score alone were 1.43, 95% CI 0.81–2.55 and 1.43, 95% CI0.93–2.2, respectively. When adjusting for confounders before ECMO support and ventilation settings (tidal volume, PEEP, respiratory rate, plateau pressure and driving pressure) and operational characteristics of ECMO (blood flow and sweep gas) at day 1 of extracorporeal support), the hazard ratio of death at 60 days decreased to 1.54 (95% CI 0.82–2.90).

**Table 2:** Outcomes

End point	COVID-19 *N* = 146	H1N1 *N* = 162	Unadjusted hazard ratio (95% CI) COVID vs H1N1^a^	Adjusted hazard ratio (95% CI) COVID vs H1N1^a,b^
Mortality at 60 days, *n* (%)	67 (46)	43 (27)	1.76 (1.17–2.64)	1.39 (0.78–2.47)
Mortality at 90 days, *n* (%)	78 (53)	48 (30)	1.63 (1.11–2.41)	1.33 (0.78–2.30)

^a^Stratified per center

^b^Adjusted for age, SOFA, SAPS II, sex, smoke, diabetes, chronic diseases, X-ray quadrants involved, rescue therapies pre-ECMO (LRM, prone position, inhaled NO), hospital LOS pre-ECMO, days of IMV pre-ECMO, ventilation setting pre-ECMO (VT/PBW, driving pressure, PaCO_2_, PaO_2_/FiO_2_)

A Cox proportional hazards model for the outcomes of 60-day mortality was also performed separately in COVID-19 and H1N1 (Additional file 1: Tables 4E and 5E). In both cohorts, patients older than sixty had the highest risk of death. In the H1N1 cohort, the burden of comorbidities and rescue therapy with inhaled nitric oxide were associated with higher risk of 60-day mortality. At 90 days, 78 patients (53%) in the COVID-19 group and 48 (30%) patients in the H1N1 group had died (adjusted hazard ratio 1.33, 95% CI 0.78–2.30 (Table 2). Compared to H1N1, COVID-19 patients had an unadjusted longer duration of ICU and hospital stay (median days 40 vs 25 and 50 vs 38, respectively: P < 0.001) and longer duration of invasive mechanical ventilation and ECMO (median days 22 vs 13 and 33 vs 25, respectively: P < 0.005) (Table 3).

**Table 3:** End points

Outcome	COVID-19 *N* = 146	H1N1 *N* = 162	*P* Value
Length of stay, (days)			
In the ICU	40 (23–78)	25 (17–41)	0.0001
In the hospital	50 (28–86)	38 (27–53)	0.0001
ECMO duration, (days)	22 (11–38)	13 (9–22)	0.0001
Duration of invasive mechanical ventilation, (days)	33 (20–62)	25 (17–43)	0.002
ECMO associated complications			
Hemorrhagic, *n (%)*	68 (47)	51 (32)	0.009
Cannula site	32 (22)	22 (14)	0.06
Airways	40 (28)	21 (13)	0.002
Gastrointestinal	9 (6)	12 (8)	0.647
Central nervous system	8 (6)	6 (4)	0.467
Mechanical, *n (%)*	38 (27)	53 (34)	0.189
Cannula thrombosis	6 (4)	4 (3)	0.420
Membrane Clotting	40 (28)	48 (31)	0.649
Pump malfunction	0 (0)	4 (3)	0.056

### Complications after ECMO cannulation

The frequency of hemorrhagic complications was higher in COVID-19 (47%) than H1N1 (31%) patients. Bleeding was more frequent in the airways, while it was similar in all other sites including the central nervous system (Table 3). There were no differences in ECMO-related mechanical complications between the two groups (Table 3).

### Time course of mechanical ventilation and ECMO operational characteristics

In both groups, tidal volume, plateau pressure, driving pressure, and respiratory rate significantly decreased over time after starting ECMO (Additional file 1: Tble 3E). However, from day 1 to 14, driving pressure was significantly higher in the COVID-19 compared to the H1N1 cohort, while PEEP was lower (Additional file 1: Table 3E). PaO_2_ was lower over time in the COVID-19 group compared to H1N1, while PaCO2 had the opposite trend. VV ECMO blood flow and sweep gas flow were similar in the two groups, except for higher blood flow at day 1 and 14 in the COVID-19 group. The daily dose of heparin was similar in the two study groups, as well as the values of aPTTr. D-dimer was higher in the COVID-19 group than in the H1N1 group at day 14 and 21, instead platelets were lower (Additional file 1: Table 3E).

## Discussion

This retrospective clinical study shows that unadjusted 60-day mortality in patients treated with VV ECMO for ARDS complicating viral pneumonia was higher in patients infected by SARS-CoV-2 than influenza A H1N1 virus. Increasing age and time to cannulation after ICU admission accounted for the excessive mortality of COVID-19 patients. These data suggest that the outcome of patients with ARDS caused by viral infection may depend on patient selection rather than the different viral etiology.

In the last 20 years, several pandemics challenged the capacity of health care systems. Severe Acute Respiratory Syndrome (SARS) in 2003 [4], influenza A H1N1 in 2009 [13], COVID-19 in 2019 [14] all had the following in common: (a) high incidence of ARDS from viral pneumonia; (b) difficulty in guaranteeing levels of assistance adequate to the severity of clinical pictures due to overcrowding in hospitals. Delivering ECMO during an outbreak of an emerging infectious disease may be demanding due to the vast technical and human resources required, along with voluminous patients’ caseloads and concerns about its benefit in a period of scarce resources [15, 16]. These issues are particularly relevant for the COVID-19 pandemic since hospital mortality, likelihood of receiving invasive mechanical ventilation and ICU length of stay have been shown to be three times higher in COVID-19 patients compared to influenza [6].

We aimed to compare outcomes of COVID-19 and H1N1 patients to understand potential differences in the severity of the two viral infections leading to ARDS. The presentation of patients with COVID-19 and H1N1 influenza requiring ECMO differed considerably before cannulation in terms of age, comorbidities and time spent before starting ECMO. Patients with influenza A(H1N1)-associated ARDS shared some similarities to those enrolled in previous observational studies regarding pre-ECMO support characteristics such as age (around 40 yrs), days of invasive mechanical ventilation before cannulation (2 days) and hospital mortality rate (30%) [2, 3, 5, 17]. Instead, COVID-19 patients were older than H1N1 patients (53 vs 47 yrs) or those enrolled in two observational studies by Schmidt and colleagues and by Extracorporeal Life Support Organization (ELSO) (53 vs 49 yrs). Moreover, 60-day mortality of COVID-19 patients was 10% higher (up to 46%) than previously reported [9, 10] and more comparable to that seen in a study by Karragiannidis and colleagues of ECMO-treated COVID-19 patients with ages ranging from 18 to 49 years [18]. The Cox model showed that the older age of COVID-19 patients was an independent risk factor for higher mortality. It is worth noting that data from the observational, registry study of ELSO and from robust meta-regression analysis highlighted increasing age (higher than 60) as the strongest predictor of dismal outcome in COVID-19 patients [9, 11, 19]. Differences in age between the two cohorts of our study may partly explain different results from an observational study of 52 patients in which both age and hospital mortality did not differ between COVID-19 and H1N1 patients [20]. In addition, not only increasing age but also days spent in the ICU before ECMO was an independent risk factor for higher mortality in COVID-19 patients compared to H1N1 patients. This finding is confirmed by a study by Karagiannidis and colleagues who found that survival also decreased according to days of mechanical ventilation prior to ECMO [18]. SOFA was not associated with increasing risk of death, a finding that has been previously published in metanalysis [11]. Among pre-ECMO rescue therapies associated with survival benefits, prone position was applied to a lower proportion of patients (76%) than in the French study (94%), and it was not associated with outcome [10]. However, we did not consider the rate of during-ECMO prone positioning that has been shown to improve survival [21]. Regardless of ARDS etiology, ECMO support allowed significant reduction in tidal volume, plateau pressure, driving pressure and respiratory rate in nearly all patients, minimizing the risk of ventilator-induced lung injury and biotrauma [22]. Except for day 1, the daily dose of heparin and values of aPTTr were similar in the two cohorts. However, COVID-19 patients had higher value of D-dimer and lower platelet counts at day14 and 21. This indicates more fibrinolysis activation in COVID 19 that could explain higher rates of hemorrhagic complications in COVID-19 that were limited to airways. Hemorrhagic stroke occurred in 4–5% of both groups. This proportion is similar to that of a large observational study involving COVID-19, but still lower than 10% and 22.7% in COVID 19 and H1N1 patients enrolled in a small study [10, 20]. In the EOLIA trial, hemorrhagic stroke only occurred in 2% of the general ARDS population [23]. Indeed, membrane clotting occurred in 30% of both groups. Whether viral pneumonia is a risk factor for coagulation activation and membrane clotting deserves further research. The current study has several limitations; firstly, patients’ outcomes were compared in a period of more than 10 years in which standard of care has changed. However, selected academic ECMO centers with high volume and experience in extracorporeal support should guarantee a high standard of care in treating both COVID and H1N1 patients. Secondly, COVID 19 patients were enrolled during the pandemia, while H1N1 patients were also enrolled outside the pandemic phase. We acknowledge that the hospital surge during the pandemia may affect patient’s outcome. Older age and longer time spent in hospital before starting ECMO are factors associated with increased mortality in COVID-19 patients. However, our approach can negate the effects of residual confounding. Unmeasured factors that may have influenced decision-making include evaluations over a telephone consult during the unprecedented pandemic strain. All ECMO centers expanded their maximum capacity; however, it is reasonable to assume that significant system load during the pandemic could have affected day-to-day decisions to offer ECMO at referring or receiving centers. Thirdly, the possibility of residual confounders such as nosocomial superinfection in determining outcomes cannot be excluded. This may be relevant since bacterial superinfections are reported to cause up to 80% of ventilator-associated pneumonia in COVID-19 patients [10]. Fourthly, indications, weaning criteria and anticoagulation regimen for ECMO were not standardized between centers, reflecting only institutional practices that—in any case -reflect the best standard of care. Moreover, all analyses were stratified by center. Fifthly, missing data were present, and this could affect the association between variables with missing data and mortality. However, the multiple imputation approach implemented in our study should have minimized the risk of bias. Sixthly, long-term outcomes of patients supported with ECMO were not available, given the retrospective design of the current study.

## Conclusions

In patients with ARDS who received ECMO, 60-day mortality was higher in cases of COVID-19 than H1N1 pneumonia. This excess in mortality could be explained by older age and longer length of hospital stay before ECMO in COVID-19 patients. These data suggest that the outcome of patients with ARDS caused by viral infection may depend on patient selection rather than the different viral etiology. Careful evaluation of patients’ characteristics prior to starting ECMO is required to identify which COVID-19 patients may derive the greatest benefit from extracorporeal support and to guide clinical decision making, especially in the context of pandemic surge.

## Supplementary Information

**Additional file 1.** Supplementary tables and figures.

## Data Availability

The datasets used and/or analyzed during the current study are available from the corresponding author upon reasonable request.

## Abbreviations

ECMO: Extracorporeal membrane oxygenation
ARDS: Acute respiratory distress syndrome
ICU: Intensive care unit
RT-PCR: Reverse transcription-polymerase chain reaction
PaO_2_: Partial pressure of arterial oxygen
PaCO_2_: Partial pressure of carbon dioxide
FiO_2_: Fraction of inspired oxygen
BMI: Body mass index
PBW: Predicted body weight
SAPS II: Simplified Acute Physiology Score II
SOFA: Sequential organ failure assessment
iNO: Inhaled nitric oxide
LRM: Lung recruitment maneuvers
PEEP: Positive end expiratory pressure
SARS-CoV-2: Severe acute respiratory syndrome coronavirus 2
ELSO: Extracorporeal Life Support Organization
aPTTr: Activated partial thromboplastin time ratio

## References

[1] Peek GJ, Mugford M, Tiruvoipati R, Wilson A, Allen E, Thalanany MM, Hibbert CL, Truesdale A, Clemens F, Cooper N (2009). Efficacy and economic assessment of conventional ventilatory support versus extracorporeal membrane oxygenation for severe adult respiratory failure (CESAR): a multicentre randomised controlled trial. Lancet.

[2] Patroniti N, Zangrillo A, Pappalardo F, Peris A, Cianchi G, Braschi A, Iotti GA, Arcadipane A, Panarello G, Ranieri VM (2011). The Italian ECMO network experience during the 2009 influenza A(H1N1) pandemic: preparation for severe respiratory emergency outbreaks. Intensive Care Med.

[3] Noah MA, Peek GJ, Finney SJ, Griffiths MJ, Harrison DA, Grieve R, Sadique MZ, Sekhon JS, McAuley DF, Firmin RK (2011). Referral to an extracorporeal membrane oxygenation center and mortality among patients with severe 2009 influenza A(H1N1). JAMA, J Am Med Assoc.

[4] Fowler RA, Lapinsky SE, Hallett D, Detsky AS, Sibbald WJ, Slutsky AS, Stewart TE, Toronto SCCG (2003). Critically ill patients with severe acute respiratory syndrome. JAMA.

[5] Zangrillo A, Biondi-Zoccai G, Landoni G, Frati G, Patroniti N, Pesenti A, Pappalardo F (2013). Extracorporeal membrane oxygenation (ECMO) in patients with H1N1 influenza infection: a systematic review and meta-analysis including 8 studies and 266 patients receiving ECMO. Crit Care.

[6] Piroth L, Cottenet J, Mariet AS, Bonniaud P, Blot M, Tubert-Bitter P, Quantin C (2021). Comparison of the characteristics, morbidity, and mortality of COVID-19 and seasonal influenza: a nationwide, population-based retrospective cohort study. Lancet Respir Med.

[7] Henry BM, Lippi G (2020). Poor survival with extracorporeal membrane oxygenation in acute respiratory distress syndrome (ARDS) due to coronavirus disease 2019 (COVID-19): Pooled analysis of early reports. J Crit Care.

[8] Roedl K, Jarczak D, Thasler L, Bachmann M, Schulte F, Bein B, Weber CF, Schafer U, Veit C, Hauber HP (2021). Mechanical ventilation and mortality among 223 critically ill patients with coronavirus disease 2019: A multicentric study in Germany. Aust Crit Care.

[9] Barbaro RP, MacLaren G, Boonstra PS, Iwashyna TJ, Slutsky AS, Fan E, Bartlett RH, Tonna JE, Hyslop R, Fanning JJ (2020). Extracorporeal membrane oxygenation support in COVID-19: an international cohort study of the Extracorporeal Life Support Organization registry. Lancet.

[10] Schmidt M, Hajage D, Lebreton G, Monsel A, Voiriot G, Levy D, Baron E, Beurton A, Chommeloux J, Meng P (2020). Extracorporeal membrane oxygenation for severe acute respiratory distress syndrome associated with COVID-19: a retrospective cohort study. Lancet Respir Med.

[11] Ramanathan K, Shekar K, Ling RR, Barbaro RP, Wong SN, Tan CS, Rochwerg B, Fernando SM, Takeda S, MacLaren G (2021). Extracorporeal membrane oxygenation for COVID-19: a systematic review and meta-analysis. Crit Care.

[12] Schmidt M, Bailey M, Sheldrake J, Hodgson C, Aubron C, Rycus PT, Scheinkestel C, Cooper DJ, Brodie D, Pellegrino V *et al*: Predicting survival after extracorporeal membrane oxygenation for severe acute respiratory failure. The Respiratory Extracorporeal Membrane Oxygenation Survival Prediction (RESP) score. *Am J Respir Crit Care Med* 2014, 189(11):1374–1382. 10.1164/rccm.201311-2023OC 24693864

[13] Investigators AI, Webb SA, Pettila V, Seppelt I, Bellomo R, Bailey M, Cooper DJ, Cretikos M, Davies AR, Finfer S (2009). Critical care services and 2009 H1N1 influenza in Australia and New Zealand. N Engl J Med.

[14] Grasselli G, Zangrillo A, Zanella A, Antonelli M, Cabrini L, Castelli A, Cereda D, Coluccello A, Foti G, Fumagalli R (2020). Baseline Characteristics and Outcomes of 1591 Patients Infected With SARS-CoV-2 Admitted to ICUs of the Lombardy Region Italy. JAMA.

[15] MacLaren G, Combes A, Brodie D (2021). What's new in ECMO for COVID-19?. Intensive Care Med.

[16] Abrams D, Lorusso R, Vincent JL, Brodie D (2020). ECMO during the COVID-19 pandemic: when is it unjustified?. Crit Care.

[17] Davies A, Jones D, Bailey M, Beca J, Bellomo R, Blackwell N, Forrest P, Gattas D, Granger E, Herkes R *et al*: Extracorporeal membrane oxygenation for 2009 influenza A(H1N1) acute respiratory distress syndrome. JAMA 2009, **302**(17):1888–1895. 10.1001/jama.2009.1535 19822628

[18] Karagiannidis C, Strassmann S, Merten M, Bein T, Windisch W, Meybohm P, Weber-Carstens S: High in-hospital mortality in COVID patients receiving ECMO in Germany - a critical analysis. *Am J Respir Crit Care Med* 2021. 10.1164/rccm.202105-1145LE PMC8534613 34283685

[19] Schmidt M, Pham T, Arcadipane A, Agerstrand C, Ohshimo S, Pellegrino V, Vuylsteke A, Guervilly C, McGuinness S, Pierard S *et al*: Mechanical ventilation management during extracorporeal membrane oxygenation for acute respiratory distress syndrome. an international multicenter prospective cohort. Am J Respir Crit Care Med 2019, **200**(8):1002–1012. 10.1164/rccm.201806-1094OC 31144997

[20] Cousin N, Bourel C, Carpentier D, Goutay J, Mugnier A, Labreuche J, Godeau E, Clavier T, Grange S, Tamion F (2021). SARS-CoV-2 versus influenza-associated acute respiratory distress syndrome requiring veno-venous extracorporeal membrane oxygenation support. ASAIO J.

[21] Giani M, Martucci G, Madotto F, Belliato M, Fanelli V, Garofalo E, Forlini C, Lucchini A, Panarello G, Bottino N *et al*: Prone positioning during venovenous extracorporeal membrane oxygenation in acute respiratory distress syndrome. a multicenter cohort study and propensity-matched analysis. *Ann Am Thorac Soc* 2021, **18**(3):495–501. 10.1513/AnnalsATS.202006-625OC 32941739

[22] Rozencwajg S, Guihot A, Franchineau G, Lescroat M, Brechot N, Hekimian G, Lebreton G, Autran B, Luyt CE, Combes A (2019). Ultra-protective ventilation reduces biotrauma in patients on venovenous extracorporeal membrane oxygenation for severe acute respiratory distress syndrome. Crit Care Med.

[23] Combes A, Hajage D, Capellier G, Demoule A, Lavoue S, Guervilly C, Da Silva D, Zafrani L, Tirot P, Veber B (2018). Extracorporeal membrane oxygenation for severe acute respiratory distress syndrome. N Engl J Med.

