# Supplementary material for: Extracorporeal membrane oxygenation for COVID-19 and influenza H1N1 associated acute respiratory distress syndrome: a multicenter retrospective cohort study
Source: Crit Care. 2022 Feb 5;26:34. doi: 10.1186/s13054-022-03906-4 (PMC8817653; doi:10.1186/s13054-022-03906-4)
Supplement: Supplementary file 1 — Additional file 1. Supplementary tables and figures. [file 13054_2022_3906_MOESM1_ESM.docx]

**Extracorporeal Membrane Oxygenation for COVID-19 and influenza H1N1 associated acute respiratory distress syndrome: a multicenter retrospective cohort study**

Vito Fanelli, MD, PhD^1,2^, Marco Giani^3^, Giacomo Grasselli^4^, Francesco Mojoli^5^, Gennaro Martucci^6^, Lorenzo Grazioli^7^, Francesco Alessandri^8^, Silvia Mongodi^5^, Gabriele Sales^1,2^, Giorgia Montrucchio^1,2^, Costanza Pizzi^9^, Lorenzo Richiardi^9^, Luca Lorini^7^, Antonio Arcadipane^6^, Antonio Pesenti^4^, Giuseppe Foti^3^, Nicolò Patroniti^10^, Luca Brazzi^1,2^, Marco V Ranieri^11^.

**ONLINE DATA SUPPLEMENT**

^1^ Department of Surgical Sciences, University of Turin, Italy.

^2^ Department of Anesthesia, Critical Care and Emergency - Città della Salute e della Scienza Hospital – University of Turin, Italy.

^3^ Department of Emergency and Intensive care, ASST Monza, School of Medicine and Surgery, University of Milano-Bicocca, Monza, Italy.

^4^ Department of Anesthesia, Critical Care and Emergency, Fondazione IRCCS Ca' Granda Ospedale Maggiore Policlinico, Milan, Italy.

^5^ Anesthesia and Intensive Care, Fondazione IRCCS Policlinico San Matteo, Università degli Studi di Pavia, Italy.

^6^ IRCCS-ISMETT (Istituto Mediterraneo per i Trapianti e Terapie ad alta specializzazione), Department of Anesthesia and Intensive Care, Palermo, Italy.

^7^ Department Emergency and Critical Area – ASST Papa Giovanni XXIII Bergamo, Italy.

^8^ Department of Anesthesia and Intensive Care Medicine, "Sapienza" University of Rome, Policlinico Umberto I, Rome, Italy.

^9^ Department of Medical Sciences, University of Turin, Italy.

^10^ Cancer Epidemiology Unit, Città della Salute e della Scienza di Torino University Hospital and CPO-Piemonte, Turin Italy.

^11^ Anesthesia and Intensive Care, San Martino Policlinico Hospital - IRCCS for Oncology and Neurosciences, Genoa, Italy; Department of Surgical Sciences and Integrated Diagnostics [DISC], University of Genoa, Genoa, Italy.

^12^ Alma Mater Studiorum – Università di Bologna, Dipartimento di Scienze Mediche e Chirurgiche (DIMEC), Anesthesia and Intensive Care Medicine, IRCCS Policlinico di Sant’Orsola, Bologna, Italy.

**Corresponding Author:**

Vito Fanelli MD, PhD

Associate Professor

Department of Surgical Sciences, University of Turin, Italy

Department of Anesthesia, Critical Care and Emergency - Città della Salute e della Scienza Hospital – University of Turin, Italy

Corso Dogliotti 14, 10126 Torino, Italy.

Tel +39-011 633 4005 (office)

Fax +39-011 6960448

[vito.fanelli@unito.it](mailto:vito.fanelli@unito.it)

**Methods**

This multi-center retrospective cohort study was conducted at ECMO referral centers of seven Italian teaching hospitals (Table 1E) from August 22th 2009 to February 28th 2021. Centers involved in the study are part of a national ECMO network where specialists perform remote assessment, deliver advice, and consider patients against eligibility criteria for retrieval on mobile ECMO, since the 2009 influenza A H1N1 pandemic. In all centers, patients were considered eligible for VV ECMO according to shared criteria: under protective mechanical ventilation (tidal volume of 6 ml/kg to keep plateau pressure below 30 cmH_2_O), deep sedation, muscle paralysis had a ratio of partial pressure of arterial oxygen (PaO_2_) to the fraction of inspired oxygen (FiO_2_) of less than 100 [1-3] and did not respond to the prone position [4]. Patients were considered unsuitable for extracorporeal support if any of the following were present: injurious ventilation at plateau pressure > 30 cmH_2_O for more than a week, contraindication to systemic anticoagulation with heparin, chronic respiratory failure requiring oxygen therapy or non-invasive ventilation, cancer with a life expectancy of less than 5 years, moribund patient as judged by the treating physician and logistic situation in which the ECMO mobile service is not immediately available [3, 5]. All participating centers were able to perform an ECMO mobile procedure 24 h per day, 7 days a week, as previously described [1, 3]. Patients who were eligible to receive ECMO at tertiary intensive care units, were transferred to one of the referral centers [1-3]. ECMO cannulation was performed percutaneously, a blood drainage cannula was inserted into the common femoral vein, and a reinfusion cannula was inserted into the right internal jugular vein or into the common femoral vein, according to the attending physician’s decision. Pump speed and FiO_2_ on ECMO was adjusted to obtain blood-oxygen saturation of more than 90% with FiO_2_ on ventilator <60%. To minimize the risk of ventilator-induced lung injury, sweep gas was adjusted to allow a tidal volume of 4 ml/kg and plateau pressure <25 cmH_2_O and driving pressure (plateau pressure-peep) <15 cmH_2_O [1, 3]. Anticoagulation with unfractionated heparin was adjusted to target a ratio of activated partial thromboplastin time between 1.51-2.34 [1, 3].

**Statistical Analysis**

Continuous variables are presented as medians and interquartile ranges (IQR). Categorical variables are presented as counts and percentages. We compared medians and percentages between COVID-19 and H1N1 groups with rank sum and chi square tests, respectively. We estimated the distribution of mortality (for any cause) over 60 days by Kaplan-Meier curve and we checked if hospital discharge was a competing event by calculating the cumulative incidence function using the Fine and Grey model. We fit a Cox proportional hazards model for the outcomes of 60- and 90-day mortality accounting for patient characteristics and treatment factors before ECMO commencement. We censored patients who were still hospitalized at the time of the last database update. The Cox model estimated the hazard ratio of death accounting for the following potential confounders categorized as shown in Table 1: age, sex, body mass index, underlying comorbidities, patient severity (SOFA), pre-ECMO hospital length of stay, rescue therapies before ECMO (lung recruiting maneuvers, prone position and inhaled nitric oxide), ventilation settings and gas exchange before ECMO. The hazard ratio of death at 60 days was estimated accounting for potential confounders such as ventilation settings (tidal volume, PEEP, respiratory rate, plateau pressure and driving pressure) and operational characteristics of ECMO (blood flow and sweep gas) at day 1 of extracorporeal support. In addition, the Respiratory ECMO Survival Prediction (RESP)-Score, which is a validated tool to predict survival for patients that receive ECMO for respiratory failure [6], was incorporated into the Cox model either by adding it to the above selected variables or by itself. Hazard ratio of death at 60 days and 90 days was estimated stratifying by center-level. Multiple imputation (50 imputed datasets) was used to account for missing values, using chained equations that fill in missing values in multiple variables iteratively. To further account for potential confounders, the propensity score of being affected by COVID-19 vs H1N1 based on all the covariates described above was calculated using a probit model, and the derived score was treated as covariate in the Cox model as a sensitivity analysis. Only subjects belonging to the common support were included both in the main multivariable Cox model adjusted for all the potential confounders and in the model adjusted for the propensity score. The common support is the area where the estimated propensity scores for COVID-19 and H1N1 patients overlap. A convenient sample size for the study was planned in order to include patients admitted to ICUs of participating centers in the study period (from August 22^th^ 2009 to February 28^th^ 2021). Statistical analyses were performed using Stata 16.1/SE (Stata Corporation, Texas, USA).

**Figure Legends**

**Figure 1E**. Causes of death of patients with COVID-19 and H1N1 associated ARDS within and after day-60.

**Table 1E**. Number of COVID-19 and H1N1 patients for each ECMO center

| **Center** | **COVID-19** | **H1N1** | **Total** |
| --- | --- | --- | --- |
| ASST Ospedale Papa Giovanni XXIII Bergamo | 12 | 1 | **13** |
| Policlinico di Milano Ospedale Maggiore \| Fondazione IRCCS Ca' Granda | 11 | 17 | **28** |
| Ospedale San Gerardo Monza | 17 | 42 | **59** |
| Istituto Mediterraneo per i Trapianti e Terapie ad Alta Specializzazione – ISMETT Palermo | 33 | 33 | **66** |
| Fondazione I.R.C.C.S. Policlinico San Matteo Pavia | 29 | 17 | **46** |
| Azienda Policlinico Umberto I Rome | 9 | 10 | **19** |
| Città della Salute e della Scienza of Turin | 35 | 42 | **77** |
| **Total** | **146** | **162** | **308** |

**Table 2E**. Number of missing data

| Variable | COVID-19  N=146 | H1N1  N=162 |
| --- | --- | --- |
| Age | 0 | 0 |
| Gender - male | 0 | 0 |
| BMI | 0 | 4 |
| Underlying comorbidities*)* |  |  |
| Obesity | 0 | 4 |
| Arterial hypertension | 0 | 0 |
| Smoking | 0 | 0 |
| Diabetes | 0 | 0 |
| Asthma | 0 | 0 |
| COPD | 0 | 0 |
| Pregnancy | 0 | 0 |
| Chronic Heart Failure | 0 | 0 |
| Chronic Liver Disease | 0 | 0 |
| Chronic Renal Failure | 0 | 0 |
| Malignancy | 0 | 0 |
| Chronic Immunosuppression | 0 | 0 |
| SOFA | 2 | 1 |
| SAPS II | 2 | 4 |
| X-ray quadrants involvement | 1 | 3 |
| Hospital days before ECMO | 0 | 0 |
| ICU days before ECMO | 1 | 0 |
| Days of IMV before ECMO | 1 | 0 |
| Rescue therapies pre-ECMO*)* |  |  |
| Lung recruitment maneuvers | 25 | 2 |
| Prone Position | 3 | 1 |
| Inhaled nitric oxide | 3 | 1 |
| Ventilation setting and ABG pre-ECMO |  |  |
| PaO_2_/FiO_2_ | 10 | 11 |
| PaCO_2_ | 14 | 21 |
| pH | 14 | 22 |
| FiO_2_ | 7 | 11 |
| PEEP | 13 | 18 |
| VT/PBW | 15 | 24 |
| RR | 15 | 29 |
| Pplat | 16 | 36 |

**Table 3E.** Time course of ventilator setting, blood gas analysis, anticoagulation and ECMO operational characteristics in COVID-19 and H1N1 groups.

|  | **COVID-19** | | | | | **H1N1** | | | | |
| --- | --- | --- | --- | --- | --- | --- | --- | --- | --- | --- |
|  | **Baseline°** | **Day 1** | **Day 7** | **Day 14** | **Day 21** | **Baseline** | **Day 1** | **Day 7** | **Day 14** | **Day 21** |
| VT/PBW, ml | 6.1(4.8-7.0)*^#^ | 4.2(3.2-5.3) | 4.3(2.6-5.1)^#^ | 3.9(2.6-4.9)^#^ | 4.2(3.3-6.0) | 6.5(5.2-7.9)* | 4.5(3.3-5.8) | 4.9(3.7-6.4) | 5.3(3.7-6.8) | 3.6(2.3-5.3) |
| RR, bpm | 24(18-30)* | 10(8-12) | 10(8-14) | 12(10-15)^#^ | 13(10-18) | 25(20-30)* | 10(8-10) | 12(9-18) | 15(10-12) | 16(10-20) |
| PEEP, cmH_2_O | 12(10-14)^#^ | 12(10-15)^#^ | 10(10-14)^#^ | 10(8-14)^§#^ | 10(8-12)^§#^ | 15(13-18) | 15(14-18) | 15(12-18) | 14(11-16)^§^ | 13(10-16)^§^ |
| FiO_2_ vent, % | 100(90-100)* | 60(50-90) | 60(50-85) | 60(50-85 | 50(45-70) | 100(100-100)* | 60(40-80) | 60(50-70) | 50(44-70) | 53(40-65) |
| Pplat, cmH_2_O | 28(25-30)* ^#^ | 24(22-27)^#^ | 25(23-28)^#^ | 25(23-28) | 25(22-30) | 30(28-34)* | 26(23-29) | 27(24-30) | 26(22-29) | 28(26-29) |
| Driving Pressure, cmH_2_O | 15(12-19)* | 12(10-14) ^#^ | 12(10-16) ^#^ | 12(10-16) ^#^ | 14(10-18) | 15(12-17)* | 10(8-12) | 10(8-12) | 11(10-12) | 12(10-13) |
| pH | 7.37(7.30-7.44)*^#^ | 7.41(7.37-7.46) | 7.41(7.38-7.45)^#^ | 7.40(7.37-7.45)^#^ | 7.42(7.37-7.45)^#^ | 7.33(7.26-7.40)* | 7.41(7.37-7.45) | 7.43(7.41-7.47) | 7.43(7.40-7.46) | 7.45(7.40-7.47) |
| PaO_2_, mmHg | 64(56-82)* | 80(68-94)^#^ | 73(63-85)^#^ | 75(63-91)^#^ | 79(65-95)^#^ | 65(54-83)* | 100(84-135) | 100(75-124) | 98(79-128) | 95(82-129) |
| PaCO_2_, mmHg | 55(45-64)* | 48(43-55)^#^ | 47(41-52) | 47(43-53) | 46(38-52) | 56(46-70)* | 45(31-51) | 45(40-50) | 45(40-50) | 45(40-50) |
| HCO3, mEq/L | 30.2(25.6-34.8) | 31.6(27.5-34.4)^#^ | 29.9(27.0-32.3) | 28.5(24.9-33.0) | 28.3(24.4-31.0)^§^ | 29.1(23.9-34.7) | 28.3(26.2-32.0) | 29.4(27.2-32.0) | 29.0(26.1-32.6) | 30.0(27.2-33.5) |
| FiO_2_ membr, % |  | 100(80-100) | 100(70-100)^#^ | 100(70-100)^#^ | 100(70-100)^#^ |  | 100(80-100)^ | 80(50-100) | 70(50-100) | 60(50-100) |
| BF, L/min |  | 4.0(3.5-4.5) | 4.0(3.5-4.7)^#^ | 3.8(3.4-4.7)^#^ | 3.8(3.1-4.5) |  | 3.8(3.2-4.5)^ | 3.6(3.1-4.0) | 3.4(2.9-4.0) | 3.6(3.0-3.9) |
| SG, L/min |  | 4.0(3.5-6.0)^ | 6.0(4.5-7.5) | 6.5(5.5-8.0)^#^ | 6.0(5.0-8.0) |  | 4.0(3.0-5.0)^ | 5.5(4.0-7.0) | 5.0(3.5-6.5) | 6.0(3.5-8.0) |
| Heparin, UIx10^3^/day |  | 25312(18110-33600)# | 28305(17670-38400) | 23995(9576-31200) | 20400(6720-25695) |  | 20065(9760-26195)^ | 27000(18240-35000) | 29500(15600-36036) | 21204(11600-27741) |
| aPTTr | 1.12(0.95-1.5)* | 1.28(1.04-1.8)^#^ | 1.5(1.27-1.77) | 1.43(1.18-1.79) | 1.49(1.14-1.8) | 1.27(1.08-1.79)* | 1.75(1.3-2.65) | 1.47(1.23-1.73) | 1.52(1.3-1.77) | 1.4(1.24-1.72) |
| PLTs, x10^3^/ml | 224(159-307)^#^ | 206(151-280)^#^ | 142(79-186)^§^ | 129(70-186)^§#^ | 133(94-179)^§#^ | 149(99-213) | 150(106-209) | 147(94-207) | 173(108-251) | 181(128-275) |
| D-dimer, | 2364(1311-4544) | 3011(1436-4831) | 5679(3213-16761)^§^ | 11387(4318-19770)^§#^ | 10658(3250-19370)^§#^ | 2606(655-5095) | 2766(905-4036) | 5417(2649-11150)^§^ | 4066(2938-8823)^§^ | 3855(2565-8330) |

° Baseline refers to the time before ECMO commencement. *p<0.05 Baseline vs other time points. #p<0.05 COVID-19 vs H1N1. §p<0.05 vs Baseline.

**List of abbreviations**. VT: tidal volume. PBW: predicted body weight. RR: respiratory rate. PEEP: positive end expiratory pressure. FiO_2_ vent: inspired oxygen fraction on the ventilator. Pplat: plateau pressure. PaO_2_: arterial pressure of oxygen. PaCO_2_: arterial pressure of carbon dioxide. FiO_2_ membr: inspired oxygen fraction on the membrane lung. BF: blood flow. SG: sweep gas. aPTTr: activated partial thromboplastin time ratio. PLTs: number of platelets.

**Table 4E.** Cox model for variables associated with 60-day mortality in COVID-19 patients

| **Variable** | **Adjusted hazard ratio (95% CI)^a^** | **p value** |
| --- | --- | --- |
| **Age. years^b^** |  |  |
| 40-50 | 0.66 (0.12-3.63) | 0.63 |
| 51-60 | 1.26 (0.27-5.94) | 0.77 |
| >60 | 2.02 (0.4-10.08) | 0.39 |
| **Body mass index^c^** |  |  |
| 25-30 | 0.77 (0.3-2.02) | 0.6 |
| >30 | 0.84 (0.33-2.13) | 0.72 |
| **Sex, male** | 0.93 (0.4-2.16) | 0.87 |
| **Smoking** | 0.49 (0.19-1.26) | 0.14 |
| **Hypertension** | 1.6 (0.82-3.12) | 0.16 |
| **Diabetes** | 1 (0.46-2.19) | 0.99 |
| **Other chronic diseases^d^** | 0.82 (0.37-1.83) | 0.64 |
| **SAPS II** | 1.02 (0.99-1.07) | 0.17 |
| **SOFA** | 1.03 (0.9-1.18) | 0.63 |
| **X-ray quadrants involved>2** | 0.47 (0.13-1.72) | 0.25 |
| **Hospital days before ECMO** |  |  |
| 1-7 | 0.35 (0.07-1.82) | 0.21 |
| 8-14 | 0.42 (0.07-2.37) | 0.33 |
| >14 | 0.47 (0.08-2.63) | 0.39 |
| **Days of IMV before ECMO** | 0.98 (0.96-1) | 0.16 |
| **Rescue therapies pre-ECMO** |  |  |
| Lung recruitment manoeuvres | 0.82 (0.35-1.88) | 0.64 |
| Prone Position | 0.69 (0.33-1.4) | 0.3 |
| Inhaled nitric oxide | 1.7 (0.82-3.51) | 0.15 |
| **Ventilation setting and ABG pre-ECMO** |  |  |
| PaO_2_/FiO_2_, mmHg | 1 (0.98-1.01) | 0.97 |
| PaCO_2_, mmHg | 1 (1-1.03) | 0.02 |
| VT/PBW, ml | 1.07 (0.89-1.3) | 0.44 |
| Driving Pressure, cmH_2_O | 1.03 (0.96-1.11) | 0.36 |

^a^ Stratified per center.

^b^ age 0-40 yrs reference interval

^c^ body mass index 0-25 reference interval

^d^ Other chronic diseases include asthma, COPD, pregnancy, chronic heart failure*,* chronic liver disease, chronic renal failure, malignancy, chronic immunosuppression**.**

List of abbreviations. SOFA: Sequential Organ Failure Assessment. SAPS II: Simplified Acute Physiology Score II. IMV: invasive mechanical ventilation. PaO_2_/FiO_2_: ratio between arterial pressure and inspired fraction of oxygen. PaCO_2_: arterial pressure of carbon dioxide. VT/PBW: tidal volume divided by predicted body weight.

**Table 5E.** Cox model for variables associated with 60-day mortality in H1N1 patients

| **Variable** | **Adjusted hazard ratio (95% CI)^a^** | **p value** |
| --- | --- | --- |
| **Age. years^b^** |  |  |
| 40-50 | 3.02 (0.69-13-3) | 0.14 |
| 51-60 | 1.94 (0.47-7.99) | 0.36 |
| >60 | 7.35 (1.57-34.37) | 0.01 |
| **Body mass index^c^** |  |  |
| 25-30 | 2.03 (0.51-8) | 0.31 |
| >30 | 3.32 (0.76-14.41) | 0.11 |
| **Sex, male** | 0.39 (0.1-1.46) | 0.16 |
| **Smoking** | 0.33 (0.1-1.07) | 0.06 |
| **Hypertension** | 1.67 (0.5-5.49) | 0.4 |
| **Diabetes** | 0.34 (0.57-2.02) | 0.24 |
| **Other chronic diseases^d^** | 5.16 (1.34-19.87) | 0.02 |
| **SAPS II** | 0.99 (0.94-1.04) | 0.92 |
| **SOFA** | 1.28 (1.05-1.57) | 0.02 |
| **X-ray quadrants involved>2** | 0.8 (1.09-5.88) | 0.83 |
| **Hospital days before ECMO** |  |  |
| 1-7 | 1.76 (0.19-15.95) | 0.61 |
| 8-14 | 1.73 (0.16-18.24) | 0.65 |
| >14 | 6.58 (0.45-95.98) | 0.17 |
| **Days of IMV before ECMO** | 1 (0.88-1.16) | 0.91 |
| **Rescue therapies pre-ECMO** |  |  |
| Lung recruitment manoeuvres | 0.95 (0.33-2.69) | 0.92 |
| Prone Position | 0.44 (0.12-1.59) | 0.21 |
| Inhaled nitric oxide | 9.5 (2.45-36.83) | 0.001 |
| **Ventilation setting and ABG pre-ECMO** |  |  |
| PaO_2_/FiO_2_, mmHg | 1 (0.98-1.02) | 0.56 |
| PaCO_2_, mmHg | 1 (0.99-1.04) | 0.22 |
| VT/PBW, ml | 0.99 (0.8-1.23) | 0.98 |
| Driving Pressure, cmH_2_O | 0.95 (0.84-1.09) | 0.48 |

^a^ Stratified per center.

^b^ age 0-40 yrs reference interval

^c^ body mass index 0-25 referecme interval

^d^ Other chronic diseases include asthma, COPD, pregnancy, chronic heart failure*,* chronic liver disease, chronic renal failure, malignancy, chronic immunosuppression**.**

List of abbreviations. SOFA: Sequential Organ Failure Assessment. SAPS II: Simplified Acute Physiology Score II. IMV: invasive mechanical ventilation. PaO_2_/FiO_2_: ratio between arterial pressure and inspired fraction of oxygen. PaCO_2_: arterial pressure of carbon dioxide. VT/PBW: tidal volume divided by predicted body weight.

**Figure 1E**

**Bibliography**

1. Patroniti N, Zangrillo A, Pappalardo F, Peris A, Cianchi G, Braschi A, Iotti GA, Arcadipane A, Panarello G, Ranieri VM *et al*: **The Italian ECMO network experience during the 2009 influenza A(H1N1) pandemic: preparation for severe respiratory emergency outbreaks**. *Intensive Care Medicine* 2011, **37**(9):1447-1457.

2. Peek GJ, Mugford M, Tiruvoipati R, Wilson A, Allen E, Thalanany MM, Hibbert CL, Truesdale A, Clemens F, Cooper N *et al*: **Efficacy and economic assessment of conventional ventilatory support versus extracorporeal membrane oxygenation for severe adult respiratory failure (CESAR): a multicentre randomised controlled trial**. *Lancet* 2009, **374**(9698):1351-1363.

3. Combes A, Hajage D, Capellier G, Demoule A, Lavoue S, Guervilly C, Da Silva D, Zafrani L, Tirot P, Veber B *et al*: **Extracorporeal Membrane Oxygenation for Severe Acute Respiratory Distress Syndrome**. *N Engl J Med* 2018, **378**(21):1965-1975.

4. Guerin C, Reignier J, Richard JC, Beuret P, Gacouin A, Boulain T, Mercier E, Badet M, Mercat A, Baudin O *et al*: **Prone positioning in severe acute respiratory distress syndrome**. *The New England Journal of Medicine* 2013, **368**(23):2159-2168.

5. Ramanathan K, Antognini D, Combes A, Paden M, Zakhary B, Ogino M, MacLaren G, Brodie D, Shekar K: **Planning and provision of ECMO services for severe ARDS during the COVID-19 pandemic and other outbreaks of emerging infectious diseases**. *The lancet Respiratory medicine* 2020, **8**(5):518-526.

6. Schmidt M, Bailey M, Sheldrake J, Hodgson C, Aubron C, Rycus PT, Scheinkestel C, Cooper DJ, Brodie D, Pellegrino V *et al*: **Predicting survival after extracorporeal membrane oxygenation for severe acute respiratory failure. The Respiratory Extracorporeal Membrane Oxygenation Survival Prediction (RESP) score**. *Am J Respir Crit Care Med* 2014, **189**(11):1374-1382.
